# Paralysis in the Course of Pneumonia

**Published:** 1895-05

**Authors:** 


### PARALYSIS IN THE COURSE OF PNEUMONIA.

The fact that hemiplegia may occur during pneumonia has already been pointed out by M. Lepine. Professor Bozzolo, of Turin, in a clinical lecture, has recently studied an interesting case and endeavored to explain its etiology.

The patient was a man, 60 years of age, neither an alcoholic nor syphilitic subject, but very nervous, who entered the hospital on account of a double pleurisy, dry upon the left and serous upon the right side, productive of but little trouble. He was, however, placed in a ward with several patients suffering from pneumonia and contracted that disease. He presented all the signs of acute hepatization of the lung. On the third day the temperature suddenly fell below 37° C. (98.6° F); the patient was attacked by vomiting, strabismus, rigidity of the neck, and acute delirium. There next supervened a right-sided flaccid hemiplegia, soon followed by contractures. He died fifteen days later.

It might have been supposed that the man had succumbed to a meningitis of pneumococcous origin. This meningitis, however, ordinarily extends to the spine, but a puncture made several days before death at the level of the cauda equina gave negative results to bacteriological examination. Furthermore, the autopsy revealed the absence of any cerebral alteration. There was no thrombosis, embolism, nor hæmorrhage. There was, however, in the fissure of Rolando of the left side an œdematous thickening of the meninges, and a slight atrophic process at the level of the ascending parietal lobe. There was nothing in the gray centres. The lung was hepatized and œdematous.

In the presence of these facts how can the hemiplegia be explained? The theory of a definite lesion once disproved, and the idea of hysteria eliminated, intoxication of the cerebral cells by the toxins of the pneumococcus might be suspected. If, however, this intoxication would explain the delirium, it fails to explain the localized motor troubles. Again, in this case the pneumococcus was not found. There remain, then, two causes which are studied by M. Bozzolo. The one is general and was first indicated by Lepine. It consists in the alteration of the circulation, which might produce nutritive troubles in the brain. The other is local, and relates to the compression which might be made in the fissure of Rolando by the œdema revealed by the autopsy,—an œdema analogous to those which Chantemesse has shown in certain hemiplegias dependent upon Bright's disease.—*Le Bulletin Medical.*

